# Supplementary material for: Individual neuronal subtypes control initial myelin sheath growth and stabilization
Source: Neural Dev. 2020 Sep 28;15:12. doi: 10.1186/s13064-020-00149-3 (PMC7523326; doi:10.1186/s13064-020-00149-3)
Supplement: Supplementary file 1 — Additional file 1: Figure S1. Characterization of transgenic reporter lines used. Figure S2. Ablation removes posterior reticulospinal axon segments without altering the number of CoPA and RB local spinal cord axons. Figure S3. Effects of reticulospinal ablation on posterior spinal cord glia. [file 13064_2020_149_MOESM1_ESM.pdf]

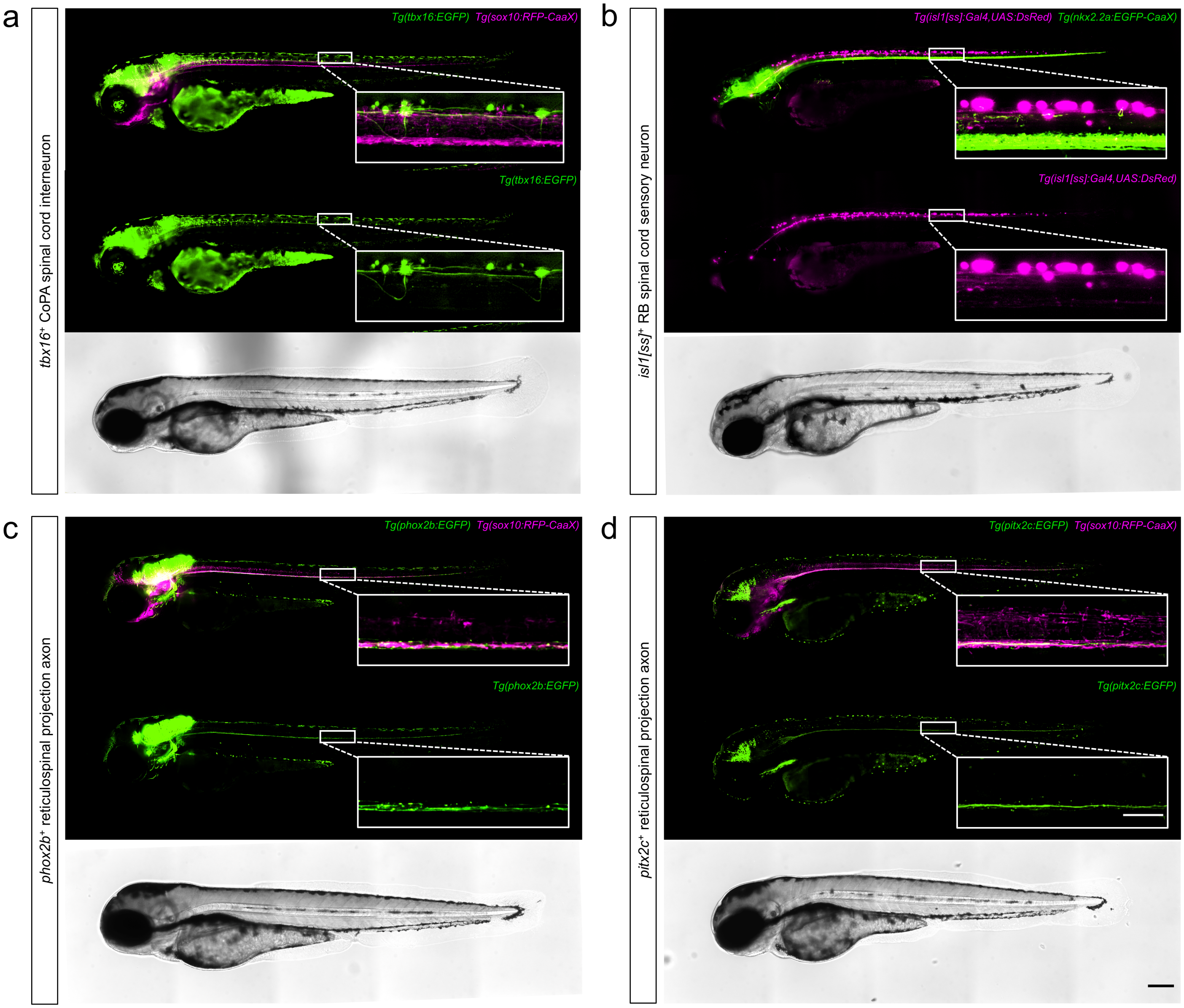

Figure S1. Characterization of transgenic reporter lines used. **a-d** Lateral view images of the larval spinal cord show an overview of transgenic reporters (indicated) used to mark defined axon subtypes or oligodendrocyte membranes. Note that *tbx16*$^+$ CoPA and *isl1[ss]*$^+$ RB axons project from spinal neurons, whereas the cell bodies of *pitx2c*$^+$ and *phox2b*$^+$ reticulospinal neurons are located in the brain. Images are tiled confocal maximum z-projections with dorsal up and anterior left. Scale bars = 200 µm (full body) and 50 µm (inset).

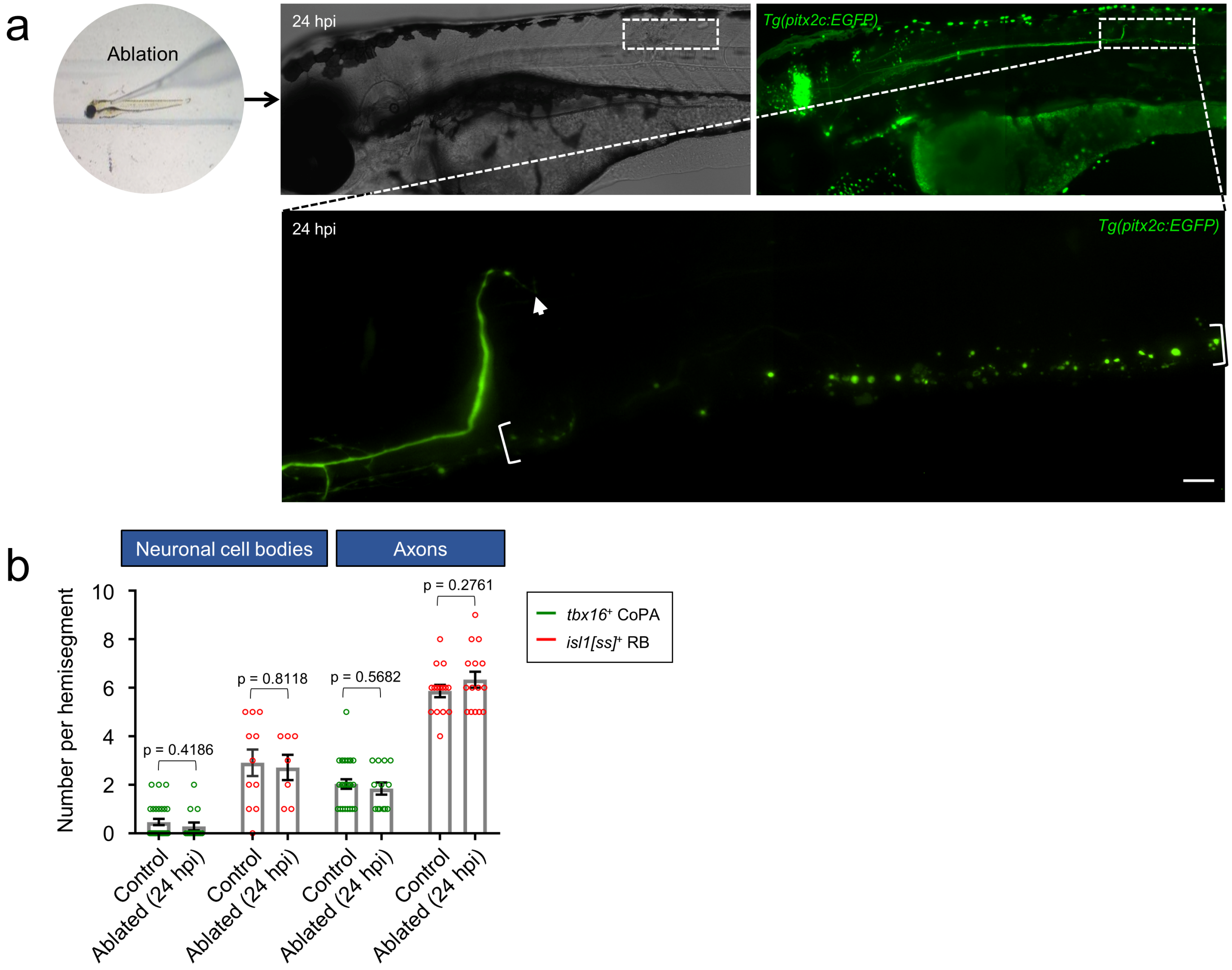

Figure S2. Ablation removes posterior reticulospinal axon segments without altering the number of CoPA and RB local spinal cord axons. **a** Anterior injury (somites 6-7) performed on a 72 hpf larva with a 60 µm glass capillary needle. Lateral view images of the larval spinal cord acquired at 24 hpi (96 hpf) show severed *pitx2c*$^{+}$ descending reticulospinal axons and Wallerian degeneration distal to the ablation site (bracketed). Images are tiled confocal maximum z-projections with dorsal up and anterior left. Scale bar = 10 µm. **b** Quantification of *tbx16*$^{+}$ CoPA and *isl1[ss]*$^{+}$ RB neuron cell bodies and axons in control (non-injured siblings) and ablated (24 hpi, 96 hpf) larvae. For neuron cell body counts, n (larvae) = 28, 14 (*tbx16*$^{+}$ control, ablated); 11, 6 (*isl1[ss]*$^{+}$ control, ablated) and for neuron axon counts, n (larvae) = 27, 13 (*tbx16*$^{+}$ control, ablated); 15, 15 (*isl1[ss]*$^{+}$ control, ablated). Error bars represent the mean ± SEM. Reported p-values were generated using a two-tailed, unpaired t-test to compare groups at matched time points.

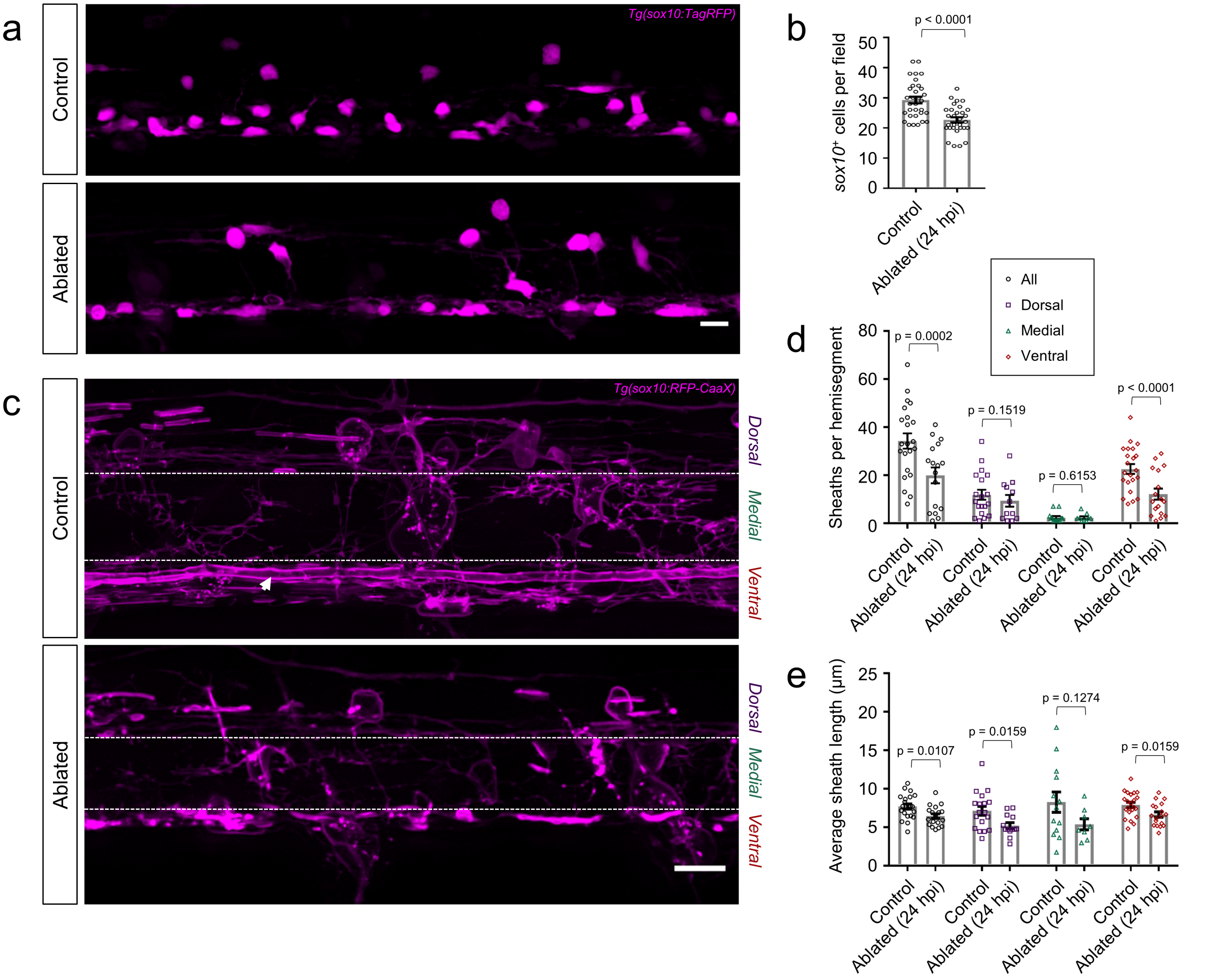

Figure S3. Effects of reticulospinal ablation on posterior spinal cord glia. **a** Lateral view images of the posterior spinal cord show oligodendrocyte-lineage cell bodies marked by *Tg(sox10:TagRFP)* in control (non-injured siblings) and ablated larvae (24 hpi, 96 hpf). **b** Quantification of oligodendrocyte-lineage cell bodies shows the number of *sox10:TagRFP*$^+$ oligodendrocyte-lineage cells per field (spinal cord hemisegment). n (larvae) = 32 (control); 31 (ablated). **c** Lateral view images of the posterior spinal cord show myelin sheaths marked by *Tg(sox10:RFP-CaaX)* in control and ablated larvae (24 hpi, 96 hpf). Dashed white lines demarcate dorsal, medial, and ventral domains (indicated). **d-e** Quantification of myelin sheaths **(d)** and average myelin sheath lengths **(e)**. Scatter plot points represent the number of *sox10:RFP-CaaX*$^+$ myelin sheaths per spinal cord hemisegment **(d)** and average sheath length per animal **(e)** reported as a total and by domain (dorsal, medial, and ventral). For **d-e**, sheaths wrapping the Mauthner axon (Supplemental Fig. 3c arrowhead) were excluded from data. For sheath counts **(d)**, n (larvae) = 22, 17 (total control, total ablated); 19, 12 (dorsal control, dorsal ablated); 13, 9 (medial control, medial ablated); 22, 17 (ventral control, ventral ablated) and for average sheath lengths **(e)**, n (larvae) = 22, 19 (total control, total ablated); 19, 14 (dorsal control, dorsal ablated); 13, 9 (medial control, medial ablated); 22, 19 (ventral control, ventral ablated). All images are tiled confocal maximum z-projections with dorsal up and anterior left. Scale bars = 10 µm. For all graphs and statistical comparisons, error bars represent the mean ± SEM. Reported p-values were generated using a two-tailed, unpaired t-test to compare groups at matched time points.
